# Preparation of Multiwall Carbon Nanotubes Embedded Electroconductive Multi-Microchannel Scaffolds for Neuron Growth under Electrical Stimulation

**DOI:** 10.1155/2020/4794982

**Published:** 2020-04-12

**Authors:** Zhenhui Liu, Maimaiaili Yushan, Yamuhanmode Alike, Yanshi Liu, Shuo Wu, Chuang Ma, Aihemaitijiang Yusufu

**Affiliations:** Department of Microrepair and Reconstruction, The First Affiliated Hospital of Xinjiang Medical University, Urumqi, Xinjiang, Uygur Autonomous Region, China

## Abstract

**Objectives:**

To prepare the conductive MWCNT (multiwall carbon nanotube)-agarose scaffolds with multi-microchannel for neuron growth under electrical stimulation.

**Methods:**

The scaffolds were produced by gradient freeze and lyophilization methods. The synthesized materials were characterized by SEM and near-infrared spectroscopy, and their microstructure, swelling-deswelling, conductivity, biocompatibility, and shape memory behavior were measured. A three-dimensional culture model by implanting cells into scaffolds was built, and the behaviors of RSC96 cells on scaffolds under electrical stimulation were evaluated.

**Results:**

The addition of MWCNT did not affect the pore composition ratio and shape memory of agarose scaffolds, but 0.025% wt MWCNT in scaffolds improved the swelling ratio and water retention at the swelling equilibrium state. Though MWCNTs in high concentration had slight effect on proliferation of RSC96 cells and PC12 cells, there was no difference that the expressions of neurofilament of RSC96 cells on scaffolds with MWCNTs of different concentration. RSC96 cells arranged better along the longitudinal axis of scaffolds and showed better adhesion on both 0.025% MWCNT-agarose scaffolds and 0.05% MWCNT-agarose scaffolds compared to other scaffolds.

**Conclusions:**

Agarose scaffolds with MWCNTs possessed promising applicable prospect in peripheral nerve defects.

## 1. Introduction

The repair and reconstruction of peripheral nerve defects caused by severe trauma, tumor excision, and congenital malformation have been a great clinic problem when gaps exceed 25 mm, because of limited resource and unsatisfactory results of autologous nerve autografting [1–4]. Regenerated nerves with anatomical appearance could not conduct the normal bioelectric signal, which could significantly affect the recovery of nerve function seriously [5]. Thus, strategies remain to be identified to improve the rate and function of nerve regeneration.

Synthetic composites can be a promising tool to guide axonal regeneration when supplying neurotrophic and/or cellular support simultaneously. Carbon nanotubes (CNTs) are increasingly used as biomedical material due to their excellent mechanical and electrical properties and high stability [6, 7]. CNTs can endow synthetic composites with good biocompatibility [8, 9], shape memory, mechanical properties [10], photothermal conversion ability, antibacterial properties [11], and conductivity which can simulate electrical conduction to guide the growth of nerve cells and promote myelination [12, 13], providing a new strategy for clinical peripheral nerve regeneration and functional reconstruction. However, CNTs were usually used as a component of composite materials, due to cytotoxicity of high concentration of carbon nanomaterials [7].

The use of agarose, with good biocompatibility and biodegradability, is in increasing expansion to satisfy different needs in bioengineering. As a saccharide polymer derived from seaweed, agarose is often used as a substrate for cell growth and bioengineering such as three-dimensional tissue growth, gene therapy, drug delivery and controlled release, and clinical application for its capacity to carry drugs and cells [14–25]. Combined with other materials to form composite materials, agarose is used to promote regeneration of skin, bone, cartilage, and nerve [22, 26–28]. However, agarose has poor electrical conductivity.

In this study, we reported the use of multiwall carbon nanotubes (MWCNTs) to enhance the electrical conductivity and biological performance of agarose scaffold. Novel MWCNT-agarose scaffolds with multi-microchannels were synthesized. The pore distribution characteristics, swelling-deswelling behaviors, conductivity, biocompatibility, and shape memory of the scaffolds were evaluated. The suitability of scaffolds loaded with RSC96 cells for neural tissue engineering and electrical stimulation system was also investigated.

## 2. Materials and Methods

### 2.1. Fabrication of MWCNT-Agarose Scaffolds

**Figure 1:**
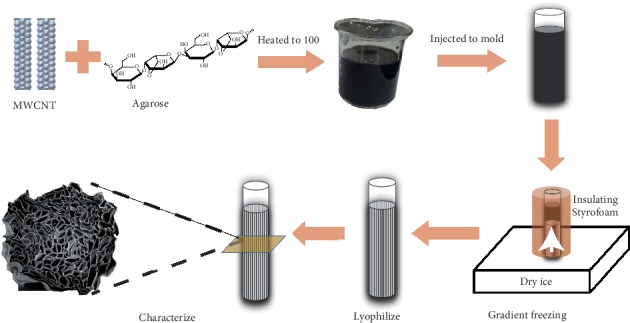
Schematic illustration of the fabrication of MWCNT-AGAROSE scaffold [21, 29].

The agarose solution, composed of 3% wt agarose (Biowest, Spain) and distilled water, was heated to 100°C until completely dissolved, injected to cylinder mold (4.6 mm diameter × 60 mm long) while removed bubbles with a vacuum drier, and then allowed to cool to be solid [21]. The MWCNTs water suspension (XFM 31) was purchased from Nanjing XFNANO Materials Technology (Nanjing, China). The MWCNTs are 5–15 nm in inner diameter and less than 10 *μ*m in length. The scanning electron microscope (SEM) and transmission electron microscopy (TEM) images and further information can be found at XFNANO Inc. For the MWCNT-contained agarose solution, MWCNTs were blended before heating at concentrations of 0.025% wt, 0.05% wt, and 0.1% wt, respectively. The molds containing agarose with or without MWCNTs were placed into insulating Styrofoam except the bottom. The entire unit was placed on dry ice, and the bottom touched dry ice directly to create a uniaxial low temperature gradient for 2 h. Then the mold unit was taken out of the Styrofoam and lyophilized for 24 h. Agarose scaffolds with or without MWCNTs were removed from the mold for characterization.  Figure 1 showed the fabrication process of the scaffolds.

### 2.2. Characterization of MWCNT-Agarose Scaffolds

#### 2.2.1. Microstructure Analysis

Cylindrical scaffolds were sectioned into 1 mm thick samples in transverse planes and longitudinal planes by stainless-steel microtome blade for morphological observation. An SEM (HITACHI SU8010) was used to observe the transverse planes, and a stereomicroscope (Leica, M205, Germany) was applied to record the longitudinal planes of those scaffolds. Gold was sputtered onto the surface of scaffolds for 10 s prior to the imaging.

Near-infrared spectroscopy (NIRQuest, ocean optics, USA) was used to analyze the chemical composition of linear agarose scaffold with different concentration of MWCNTs. The excitation wavelength ranged from 900 cm^−1^ to 2000 cm^−1^.

The hydrated samples were placed on slides to be visualized by an LSM800 confocal microscope (Zeiss), and the area of the pores was directly measured by the Axio-Imager software (Zeiss) to calculate the proportion of the pore area to the total area (porosity) and analyze the proportion of each kind of pore size to the total pore numbers. Three different samples were randomly selected from each kind of scaffolds.

#### 2.2.2. Swelling and Deswelling

The swelling behavior of linear agarose scaffolds with or without MWCNT was observed for 2580 min at a temperature of 25°C and a relative humidity of 65%. The freeze-dried scaffolds were weighed, then soaked in distilled water, and removed at a certain interval. After surface water was absorbed with filter paper, the scaffolds were weighed immediately [10, 30, 31]. There were 5 samples of each scaffold, and each sample was measured 3 times to calculate the swelling rate (*SR*) by the Equation (1)

(1) $$\begin{array}{c} SR=\frac{Ws-Wd}{Wd}\times 100\%, \end{array}$$

where *SR*, *Ws*, and *Wd* are the swelling rate of scaffolds at a certain time, the weight of scaffolds at a certain time, respectively, and the weight of dry scaffolds.

After the scaffolds reached swelling equilibrium at the temperature of 25°C and the relative humidity of 65%, the excess surface water was removed and weighed at a certain time interval to evaluate the swelling trend of the scaffolds. The measurement for each scaffold was an average of three times to calculate the water retention (*WR*) by Equation (2)

(2) $$\begin{array}{c} WR=\frac{Wt-Wd}{\text{We}-Wd}\times 100\%, \end{array}$$

where *Wt* is the weight of scaffold at a certain time of deswelling and *We* is the weight of swollen scaffold at equilibrium state [31, 32].

#### 2.2.3. Conductivity of Scaffolds

The electrical conductivity of scaffolds with or without MWCNT was measured at equilibrium state of PBS absorption at room temperature. The swelling scaffolds were placed between two copper electrodes and measured with a conductivity meter (UT61E) [33]. According to Equation (3), the resistance *ρ* in (*Ω*. m) was calculated. [34]

(3) $$\begin{array}{c} R=\rho \frac{l}{A}, \end{array}$$

where *R* is resistance of the scaffolds, *l* is length, and *A* is cross-sectional area, respectively.

The conductivity was calculated by using Equation (4)

(4) $$\begin{array}{c} \sigma =\frac{1}{\rho }m\text{ho}.m^{-1}. \end{array}$$

#### 2.2.4. Shape Memory of Scaffolds

The scaffolds which had reached the balance of water absorption reached deswelling equilibrium at room temperature and then were soaked in deionized water. The shape changes were observed by a digital video recorder (SONY) [10].

### 2.3. In Vitro Biocompatibility Study

The cylindrical scaffolds with a diameter of 4.6 mm were sliced into disc samples with a height of 2 mm. The samples were sterilized in 95% ethanol for 2 h, soaked in PBS overnight, then vacuum-dried for later use, and prewetted with 37°C complete medium before coculture with cells [35].

The RSC96 cells purchased from the ZhouQiaoXinZhou Biotechnology Co. Ltd (Shanghai, China) were cultured in DMEM (Gibco) supplemented with 10% fetal bovine serum (FBS, Gibco) and 100 *μ*g/ml streptomycin and 100 U/ml penicillin (BI) under a humidified atmosphere with 5% CO_2_ at 37°C. The RSC96 cells were seeded into the 96-well plates at a density of 3000 per well, and the prewet scaffolds were transferred into the corresponding wells at the same time. The cells in each group were cultured for 1 d, 3 d, and 5 d with five samples per group at each time point. RSC96 cells were observed in different culture conditions by a phase contrast microscope.10 *μ*l CCK8 solution (Dojindo, Kumamoto, Japan) was added to each well and incubated for 2 h at 37°C and then the absorbance OD value at 450 nm was analyzed by Thermo Scientific Multiskan GO Spectrophotometer (USA). RSC96 cells cultured on Glass Bottom Dish with scaffolds for 3 d were washed with PBS, fixed in 4% paraformaldehyde for 30 min, and washed with PBS again. After soaked in 0.15% Triton X-100 for 10 min and blocked in 10% bull serum albumin for 30 min, cells were incubated with antibodies anti-NF-H at 1 : 200 dilution at 4°C overnight, followed by incubation with secondary antibodies conjugated to Flour 488 at 1 : 200 dilution for 2 h in dark and counterstain with DAPI at 1 : 20 000 dilution for 10 min at room temperature. An inverted laser scanning confocal microscope (Leica TCS, Germany) was used to investigate the samples. Quantitative analyses of immunofluorescence were implemented by Image-Pro Plus 4.5 software (Media Cybernetics, Silver Spring, MD, USA).

The PC12 cells purchased from the ZhouQiaoXinZhou Biotechnology Co. Ltd (Shanghai, China) were cultured in DMEM/F12 (Gibco) supplemented with 10% FBS (Gibco) and 100 *μ*g/ml streptomycin and 100 U/ml penicillin (BI). PC12 cells were cocultured with scaffolds at a concentration of 5000 per well, and the OD value was determined by the CCK8 method as RSC96 cells. The scaffolds cocultured with PC12 cells were fixed by 2.5% glutaraldehyde for 4 h and dehydrated by graded ethanol. After sputtered with gold, scaffolds were imaged by SEM.

### 2.4. Three-Dimensional Cultured Model of RSC96 Cells

The cylindrical scaffolds with a diameter of 4.6 mm and a height of 12 mm were sterilized in 95% ethanol for 2 h, then soaked in PBS overnight. After vacuum-dried and incubated at 37°C for 1.5 h, scaffolds conducted deswelling-shrinking behavior. RSC96 cell suspension of 10 *μ*l was added to the scaffolds at a dense of 20 000 cells per scaffold and then absorbed into scaffolds evenly due to the shape memory property of scaffolds. After incubation for half an hour, the scaffolds were transferred to a new 6-well plate, and complete medium was added to the plate for cell growth study under a humidified atmosphere with 5% CO_2_ at 37°C. Medium was changed every other day.

The RSC96 cells on control scaffolds could be observed directly by a phase contrast microscope (Leica, Germany). After stained with DAPI at 1 : 20 000 dilution in PBS for 10 min, cells could be imaged by an inverted laser scanning confocal microscope (Leica, Germany). The scaffolds were divided into electrical stimulation (ES) group and the control group. Our customized electrical stimulator equipment consisted of the pulse stimulator and electrode attached to 6 well plate lid. ES groups were performed by applying voltage of 3-4 V, time of 30 min per day, frequency of 10 Hz, and continue waves in a Clean Bench [13].

Samples cultured for 7 d were fixed with 4% paraformaldehyde for 30 min at room temperature, embedded in paraffin and then cut into 5 *μ*m thick longitudinal sections. These sections were stained with hematoxylin for 5 min, washed with distilled water for 1 minter, stained with 0.5% eosin for 2 min, washed with distilled water, permeabilized, and mounted. Growth features of cells on scaffolds were observed using an optical microscope [36] (DM3000B; Leica).

### 2.5. Statistical Analysis

All quantitative data were presented as mean ± standard error and analyzed using SPSS software, version 23.0 (IBM, Armonk, NY, USA). Multiple group comparisons were conducted by one-way analysis of variance followed by *LSD post-hoc test.P* < 0.05 was considered as statistical significance.

## 3. Results

### 3.1. Characterization of Multi-Microchannel MWCNT-Agarose Scaffold

#### 3.1.1. Microstructure Analysis

**Figure 2:**
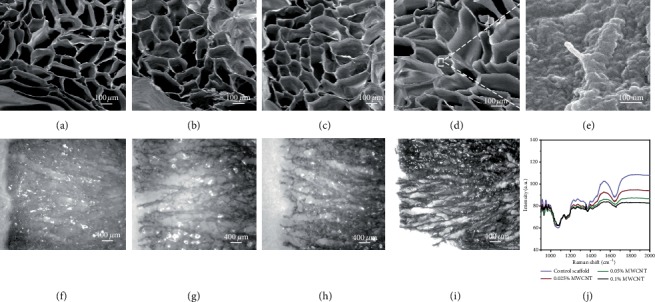
SEM images and RAMAN spectra of multi-microchannel scaffolds. (a)-(d): SEM images of multi-microchannel MWCNT-agarose scaffolds with MWCNT concentrations of 0% wt, 0.025% wt, 0.05% wt, and 0.1% wt, respectively. (e) showed scaffold with 0.1% wt MWCNTs on the surface at 30 K magnification. (f)-(i) Longitudinal section images of multi-microchannel MWCNT-agarose scaffolds with MWCNTs concentrations of 0% wt, 0.025% wt, 0.05% wt, and 0.1% wt, respectively. (j) showed the characteristic peak of MWCNTs could be observed at about 1350 cm^−1^ in 0.1% wt MWCNT-agarose scaffold.

Figure 2 showed the consistent morphology and porosity of scaffolds with or without MWCNT, presenting a microhoneycomb linear channel and penetrating the whole scaffold. It could be seen that MWCNTs and agarose fused well. Raman spectra were used to further characterize the chemical state of MWCNTs encapsulated in agarose scaffolds. Figure 2(j) showed that 0.1% wt MWCNT-agarose scaffolds showed characteristic peaks different from other scaffolds. The graphitized structure of MWCNTs had a Raman displacement called D-band at 1350 cm^−1^, and no characteristic peak was found in the MWCNT scaffolds below 0.1% wt.

**Figure 3:**
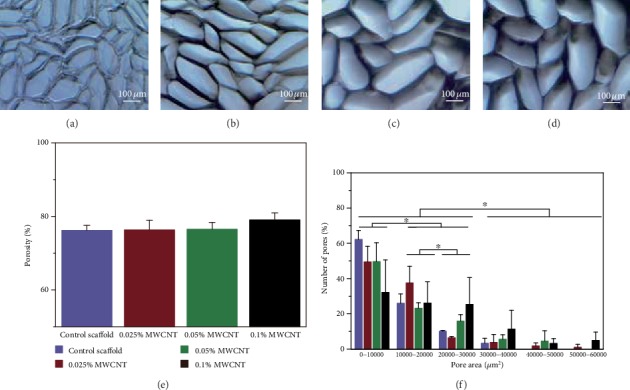
Light microscopy micrographs, porosity, and pore size distribution of multi-microchannel scaffolds. (a)-(d) showed images of linear oriented scaffolds without MWCNT (a), 0.025% wt MWCNT(b), 0.05% wt MWCNT (c), and 0.1% wt MWCNT (d). (e) showed there was no difference in porosity between scaffolds without MWCNT and with MWCNT. (f) showed the proportion of pores with different area in the respective scaffolds. ^∗^*P* < 0.05 when compared to other groups.

The porosity of scaffold without MWCNT was 76.18 ± 1.36%, scaffold with 0.025% wt MWCNTs was 76.32 ± 2.64%, scaffold with 0.05% wt MWCNTs was 76.51 ± 1.89%, and scaffold with 0.1% wt MWCNTs was (79.06 ± 1.88)%. There was no difference in the porosity among these groups (*P* < 0.05). According to the size of the pore area, pores were divided into six types: 0–10 000 *μ*m^2^, 10 000–20 000 *μ*m^2^, 20 000–30 000 *μ*m^2^, 30 000–40 000 *μ*m^2^, 40 000–50 000 *μ*m^2^, and 50 000–60 000 *μ*m^2^. The largest number proportion of pores with an area of 0–10 000 *μ*m^2^ was 48.38 ± 15.05%, followed by pores with an area of 10 000–20 000 *μ*m^2^ (28.20 ± 9.11%) and 20 000–30 000 *μ*m^2^ (14.44 ± 10.01%). These numbers of pores were higher than all the others (*P* < 0.05). There was no difference in the pore composition ratio of different pore size among the four kinds of scaffolds (see Figure 3).

#### 3.1.2. Swelling and Deswelling

**Figure 4:**
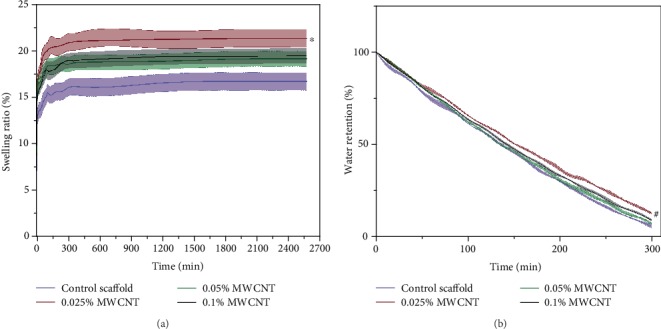
The swelling and deswelling behavior of multi-microchannel scaffolds with or without MWCNT. (a) showed that all the scaffolds reached a swelling equilibrium state at 180 min and the 0.025% MWCNT-agarose scaffolds had higher swelling ratio than others (^∗^*P* < 0.05). (b) showed the water retention of each scaffold decreased linearly with time. The 0.025% MWCNT-agarose scaffolds had higher water retention than the others after the 100 min (^#^*P* < 0.05).

The swelling behavior of scaffolds with or without MWCNT showed SR of all the scaffolds increased with time and reached a swelling equilibrium state at the 180 min. No difference was found in the increase rate of SR among groups. The 0.025% MWCNT-agarose scaffolds had higher swelling ratio than others at the equilibrium state. The deswelling behavior of scaffolds showed the water retention of each scaffold decreased linearly with time. The 0.025% MWCNT-agarose scaffolds had higher water retention than the others after the 100 min (see Figure 4).

#### 3.1.3. Conductivity of Scaffolds

**Figure 5:**
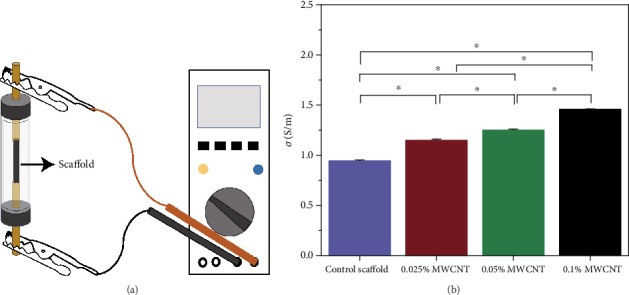
Schematic illustration of measurements of resistance and the conductivity of the multi-microchannel Scaffolds. (a) showed the measurement of resistance. (b) showed that the conductivity of scaffolds was improved gradually with increasing of MWCNTs concentration (^∗^*P* < 0.05).

The conductivity of control scaffolds, scaffolds with 0.025% MWCNT, scaffold with 0.05% MWCNT, and scaffold with 0.1% MWCNT was measured to be 0.944 ± 0.009, 1.170 ± 0.031, 1.252 ± 0.009, and 1.459 ± 0.003 S/m.  Figure 5 showed that the conductivity of scaffolds was improved gradually with increasing of MWCNTs concentration.

#### 3.1.4. Shape Memory Characterization of Scaffolds

**Figure 6:**
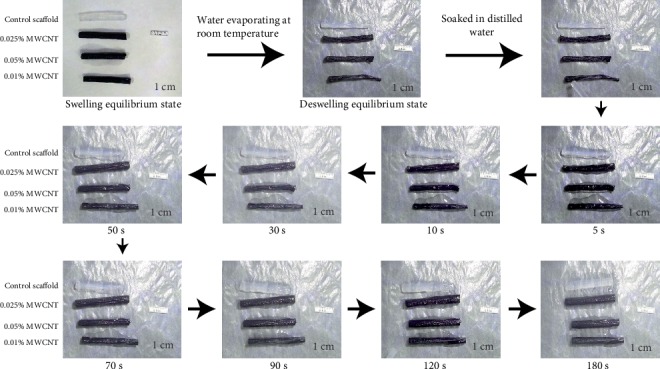
Shape memory behavior of scaffolds with or without WMCNTs.

When the scaffolds reached the balance state of deswelling at room temperature, the cylindrical structure of all the scaffolds collapsed. However, 0.1% MWCNT-agarose scaffold deformed more severely than others. After being soaked in distilled water, all the scaffolds absorbed water quickly and recovered completely to their original shape within the180 s (see Figure 6).

### 3.2. In Vitro Biocompatibility

**Figure 7:**
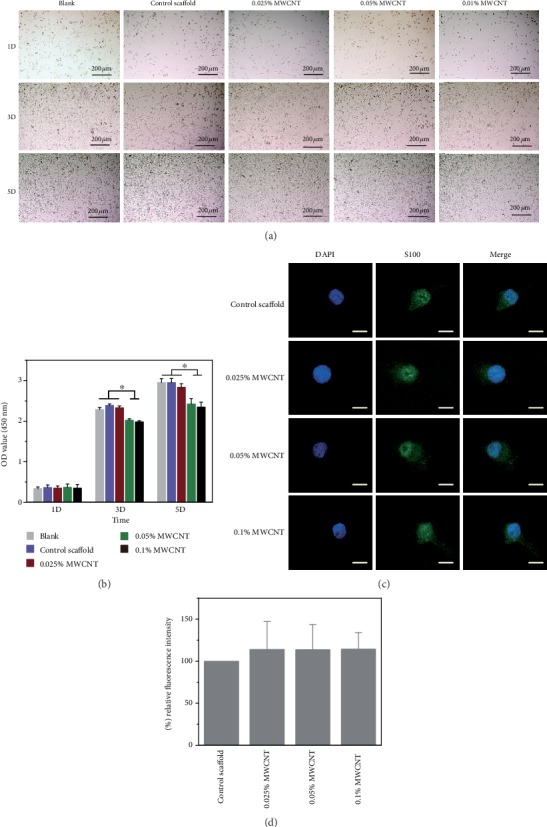
Proliferation of RSC96 cells. (a) Observation of RSC96 cell proliferation in different culture conditions under a phase contrast microscope. (b) RSC96 cell proliferation measured by CCK8 assay, and the result showed that the scaffolds with 0.05% MWCNT and 0.1% MWCNT demonstrated cytotoxicity on day 3 and day 5 (^∗^*P* < 0.05). (c) showed the neurofilament images of RSC96 cells cocultured with scaffolds by immunofluorescence. Scale bar = 50 *μ*m. (d) showed the relative expressions of NF through cell immunofluorescence intensity on scaffolds with different concentration of MWCNTs and compared with the control scaffolds after cultured for 3 days. No difference was found between any two scaffolds.

**Figure 8:**
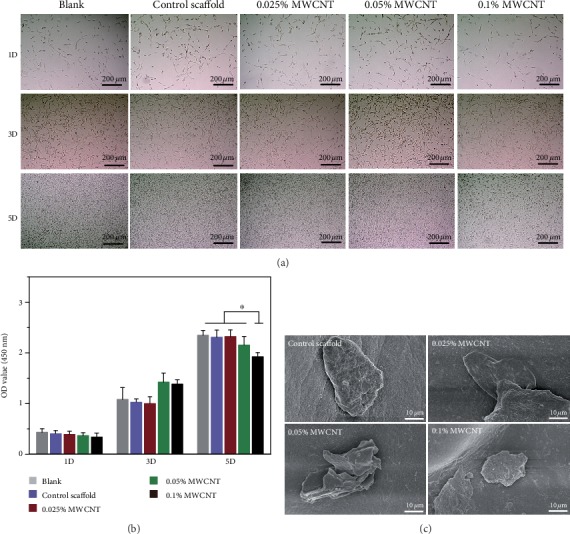
Proliferation of PC12 cells. (a) showed PC12 cells proliferation in different culture conditions under a phase contrast microscopic observation. (b) showed PC12 cells proliferation measured by CCK8 assay. The scaffolds with 0.1% MWCNT demonstrated cytotoxicity on day 5 (^∗^*P* < 0.05). (c) showed PC12 cells attached to the scaffolds by SEM.

The cytotoxicity of scaffolds to RSC96 cells and PC12 cells was evaluated by the CCK8 assay (see Figures 7 and 8). The results showed agarose scaffolds without MWCNT and with 0.025% MWCNT were not cytotoxic to RSC96 cells and PC12 cells on day 1 and day 3. Agarose scaffolds with 0.05% MWCNT and 0.1% MWCNT had cytotoxicity to SCs while only agarose scaffolds with 0.1% MWCNT had cytotoxicity to PC12 cells on day 5. However, no difference of the relative expressions of neurofilament was found between agarose scaffolds with MWCNTs of different concentration and without MWCNTs. Besides, the PC12 cells could be observed that they were attached to all the scaffolds by SEM.

### 3.3. Three-Dimensional Cultured Model of RSC96 Cells

**Figure 9:**
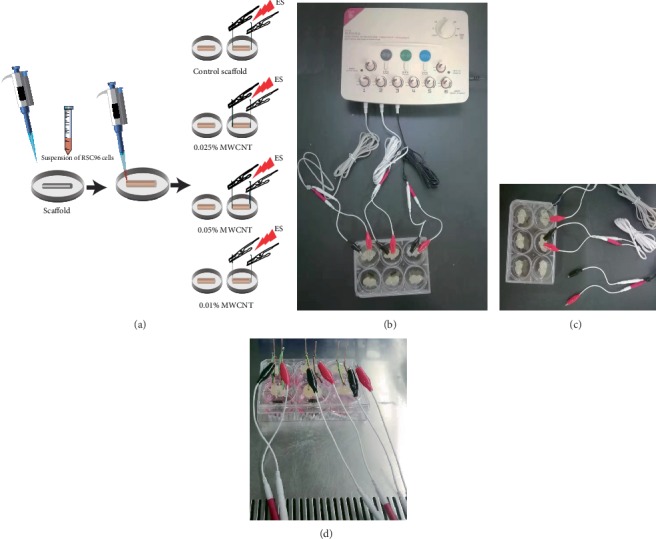
Schematic illustration of three-dimensional cultured model of RSC96 cells and a customized electrical device. (a) The scaffolds were divided into electrical stimulation (ES) group and the control group after the cell suspension was added to the deswelling scaffold and absorbed evenly. (b) and (c) showed electrode-attached 6-well plate was connected to the pulse stimulator. (d) showed cell-loading scaffolds were stimulated in a clean bench.

**Figure 10:**
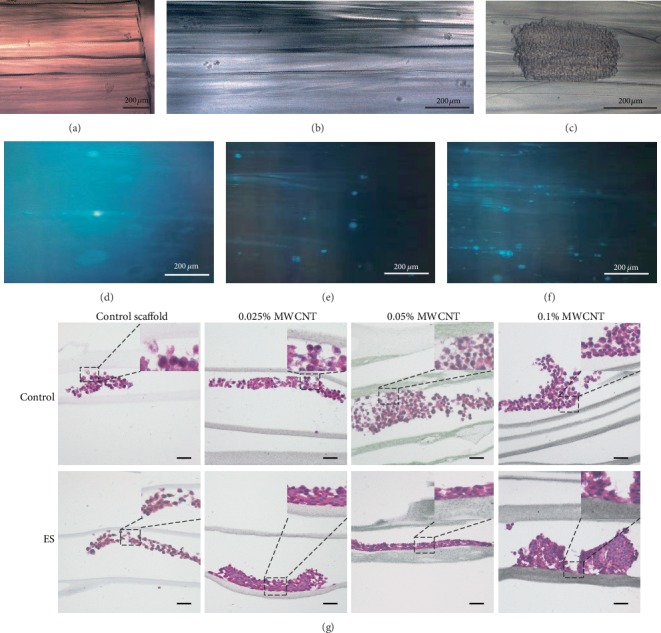
The growth behaviors of RSC96 cells on scaffolds. (a), (b), and (c) showed growth of RSC96 cells on control scaffolds observed by a phase contrast microscope. (d), (e), and (f) showed growth of RSC96 cells stained with DAPI on control scaffolds observed by an inverted laser scanning confocal microscope. (g) showed the behavior of RSC96 cells stained with HE on different culture conditions. Scale bar = 100 *μ*m.

By observing RSC96 cell growth behavior in answer to electrical stimulation, further evaluation of the scaffolds in neural tissue engineering for their potential application was done.  Figure 9 showed the integral design of the electrical stimulation installation, and Figure 10 showed the growth of RSC96 cells on the scaffolds. Accompanying by the formation of cell clumps, cells grew robustly on all the scaffolds without electrical stimulation. However, when electrical stimulation was applied on scaffolds, RSC96 cell layers arranged better along the longitudinal axis of scaffolds and showed better adhesion on both 0.025% MWCNT-agarose scaffolds and 0.05% MWCNT-agarose scaffolds than other scaffolds.

## 4. Discussion

In this study, we prepared multi-microchannel electroconductive neural scaffolds, built a three-dimensional culture model by implanting cells into scaffolds and observed that RSC96 cell layers arranged better along the longitudinal axis of electroconductive scaffolds under ES, and showed better adhesion on both 0.025% MWCNT-agarose scaffolds and 0.05% MWCNT-agarose scaffolds than other scaffolds. Our electroconductive neural scaffolds mimic the native three-dimensional extracellular matrix to provide guidance channels for nerve cells, and ES applied on conductive scaffolds can simulate physiological neuronal activity to promote the directional growth of nerve cells, which might be highly beneficial for peripheral nerve regeneration.

Although a variety of nerve conduits or scaffolds have been developed in the past decades, it remains challenging to repair long peripheral nerve gaps due to the target muscular atrophy for lack of specific signals and cues to guide the axons to the distal stump. Biomimicking concept is one option in the development of customized tissue engineering. Wu et al. prepare a 3D hybrid scaffold based on NFYs-NET layers within a hydrogel shell for mimicking the native cardiac tissue structure and demonstrated their great potential for engineering anisotropic 3D cardiac constructs [37]. They developed aligned core-shell composite biomimetic scaffolds based on aligned conductive NFYs core and hydrogel shell to mimic the hierarchically aligned 3D structure of the native nerve tissue [38]. Stokols et al. used agarose scaffolds with uniaxial channels to stimulate and guide linear axonal growth in a spinal cord injury model [22]. We evaluated anatomy-based nerve conduits in vitro and in vivo and found that multichannel nerve conduit had improved the nerve regeneration by reducing the mismatch of nerve axons in our previous research [36]. We observed that multichannel guidance contributed to limiting axonal dispersion without decreasing quantitative results of regeneration. Hollow nerve conduit was not suitable for large peripheral nerve gap for lack of axon growth-promoting substrates. Longitudinally oriented microstructure of the multi-microchannel nerve scaffolds mimics some of the natural topographical features of normal peripheral nerves and provides the framework to guide glial cell migration and axonal growth [39]. On the other hand, the multi-microchannel scaffolds can provide 3D environments which are conducive to cell attachment, migration, proliferation, and differention and helpful to generate complicated structure of newly formed tissues [40].

Electrical stimulation is well-known for promoting nerve regeneration and functional recovery [41–43]. While cellular behaviors after ES have been widely reported, the results are not yet determined. Some reports revealed ES could promote correct reinnervation and help axons cross the nerve repair site or nerve guidance channels, but it did not increase the speed of axonal elongation [44–50]. Besides, ES was believed to activate neuronal differentiation [35, 51, 52], and robust neuron regeneration was observed in vivo experiments on conductive materials with ES [53, 54]. Recent studies found enhanced neurite outgrowth following ES [41, 55]. Therefore, we observed that RSC96 cell layers arranged along the longitudinal axis under ES in Figure 10(g). This phenomenon was analogous to bands of Büngner, which was essential for nerve regeneration, indicating that our multichannel nerve scaffolds may have potential application in promoting linear axon growth through long-distance nerve defect to improve functional recovery.

As central part of the nerval biomimicking concept, the electrical conduction of nerve scaffolds is a factor that cannot be ignored. Bioelectricity plays an indispensable role in sustaining normal biological functions through tissue engineering [56]. Neurons in the nervous system are electrically excitable cells that transmit signals at a quick pace [57]. Conductive scaffolds are developed to enhance the regeneration in peripheral nerve tissue engineering. Wu et al. found that electroactive biodegradable polyurethane significantly enhanced Schwann cells myelin gene expression and neurotrophin secretion by providing suitable microenvironment [58]. Conductive scaffolds were also found to maintain PC12 cells in more active proliferation and neurite growth as well as upregulate GAP43 and SYP protein and gene expression levels [59, 60]. Carbon nanomaterial is also one of the conductive sources for biomaterials to achieve conductivity. CNTs can endow nanocomposites with excellent electrical properties, good cell adhesion, and tissue formation at a low concentration, which is determined by their nanoscale structure. Owing to an extremely high-length diameter ratio, CNTs can easily form a conductive network, which helps to improve the conductivity of nanocomposites [61–63]. In our study, we observed that the conductivity of agarose scaffolds was improved with the increased concentration of MWCNT compared with agarose scaffolds alone.

Poor functional recovery after peripheral nerve injury has been generally attributed to inability of denervated muscles to accept reinnervation and recover from denervation atrophy [64]. Ahadian et al. developed gelatin methacrylate hybrid hydrogels containing CNTs and promoted higher maturation of muscle myofibers [65]. Conductive materials have been demonstrated to promote electrical-responsive cell proliferation and enhance the myotube formation [40]. Therefore, conductive materials have shown a promising prospect in muscle tissue engineering. More studies of CNTs need to be performed to avoid atrophy of target muscle tissue.

It is well-known that agarose has good biocompatibility, and CNTs show potential in biomedicine as their low toxicity [9, 14, 15]. High concentration MWCNTs were supposed to have cytotoxicity caused by reactive oxygen in cells and damage of cell member when contacting to MWCNTs [7, 66–68]. It was reported that GelMA-coated CNTs with a concentration of 0.5 mg/ml had significative cytotoxicity [69]. In this study, we found only agarose scaffolds with 0.1% wt MWCNT have limited effect on the proliferation of RSC96 cells and PC12 cells. The cytotoxicity of CNTs coated in agarose might be reduced compared to the cytotoxicity of bare CNTs, which may explain why cells proliferation did not decrease obviously in our research.

Swelling and deswelling behaviors of scaffolds in aqueous are essential in biomedical applications because they control the diffusion and release of drugs and nutrients in tissue application [23]. The high swelling and deswelling rate of MWCNT-agarose scaffolds might be ascribed to the hydrophilicity nature of both agarose and water dispersed MWCNTs or/and changes of agarose scaffolds in spatial structure caused by MWCNTs [10, 22, 70]. As the concentration of MWCNT increases, the swelling and deswelling rate of scaffolds reduce may be attributed to the decrease of free space among agarose chains. The 0.025% MWCNT-agarose scaffolds showed higher water retention than the others, demonstrating 0.025% wt MWCNTs were near to the optimum concentration between MWCNTs and agarose. Furthermore, the water-dispersed MWCNTs showed no impact on the expressions of neurofilament, which are neuron structural element [71, 72]. This supports their application in peripheral nerve.

Scaffolds with function of shape memory are convenient for transportation, preservation, and minimally invasive approach [73]. They can regain their initial shape by recruiting extracellular fluid when agarose chains maintain their mobility and recover to their primitive position [31].

The construction of scaffolds is easy to implement, without the employment of chemical crosslinking and organic solvents; therefore, the risk of introducing toxic molecules into the scaffolds is low. The linear pores produced by gradient freeze and lyophilization constitute multi-microchannels which extend through the full length of scaffolds. However, MWCNTs dispersed in the agarose well did not alter the porosity and pore area distribution of agarose scaffold as shown in Figure 3. We evaluated pore size by pore area instead of pore diameter, which displayed the size of pores better than the previous literatures because the pores were not regular. The pore areas of the hydrated scaffolds were distributed around 10 000 *μ*m^2^, which were consistent with previous reports [22] and beneficial for cell attachment [23, 74].

In our study, scaffolds with lower MWCNT concentration had no cytotoxicity effect on cell proliferation compared to any other group on day 5 without electrical stimulation. CNTs show potential in biomedicine as their low toxicity [9]. The cytotoxicity of high concentration MWCNTs is supposed to be caused by reactive oxygen in cells and damage of cell member when contacting to MWCNTs [7, 66–68]. However, no significative cytotoxicity of GelMA-coated CNTs was found up to the CNT concentration of 0.5 mg/ml [69]. The cytotoxicity of CNTs coated in agarose might be reduced compared to the cytotoxicity of bare CNTs, which may explain why cell proliferation did not decrease obviously in our research.

## 5. Conclusion

We successfully utilized water-dispersed MWCNT and agarose to fabricate multi-microchannel electroconductive neural scaffolds by gradient freeze and lyophilization method in this study. The MWCNT-agarose scaffolds mimic the function and the three-dimensional structure of the peripheral nerve, thereby guiding linear growth of RSC96 cells under electrical stimulations. This study may provide an applicable composite material to fabricate electroconductive nerve guiding scaffolds, which can be used in the repair of the peripheral nerve defects.

## Data Availability

The data that support the findings of this study are available on request from the corresponding author. The data are not publicly available due to privacy restrictions.

## References

[1] Han B., Zhao J. Y., Wang W. T., Li Z. W., He A. P., Song X. Y. (2017). Cdc42 promotes Schwann cell proliferation and migration through Wnt/*β*-Catenin and p38 MAPK signaling pathway after sciatic nerve injury. *Neurochemical Research*.

[2] El Soury M., Gambarotta G. (2019). Soluble neuregulin-1 (NRG1): a factor promoting peripheral nerve regeneration by affecting Schwann cell activity immediately after injury. *Neural Regeneration Research*.

[3] Griffin J. W., Hogan M. V., Chhabra A. B., Deal D. N. (2013). Peripheral nerve repair and reconstruction. *The Journal of Bone and Joint Surgery-American Volume*.

[4] Grinsell D., Keating C. P. (2014). Peripheral nerve reconstruction after injury: a review of clinical and experimental therapies. *BioMed Research International*.

[5] Ryan A. J., Lackington W. A., Hibbitts A. J. (2017). A physicochemically optimized and neuroconductive biphasic nerve guidance conduit for peripheral nerve repair. *Advanced Healthcare Materials*.

[6] Fadel T. R., Fahmy T. M. (2014). Immunotherapy applications of carbon nanotubes: from design to safe applications. *Trends in Biotechnology*.

[7] Chen M., Sun Y., Liang J. (2019). Understanding the influence of carbon nanomaterials on microbial communities. *Environment International*.

[8] Zheng X., Woeppel K. M., Griffith A. Y. (2019). Soft conducting elastomer for peripheral nerve Interface. *Advanced Healthcare Materials*.

[9] Wang H., Li P., Yu D. (2018). Unraveling the enzymatic activity of oxygenated carbon nanotubes and their application in the treatment of bacterial infections. *Nano Letters*.

[10] Liu X., Song T., Chang M. (2018). Carbon nanotubes reinforced maleic anhydride-modified xylan-g-poly(N-isopropylacrylamide) hydrogel with multifunctional properties. *Materials*.

[11] Zhao X., Guo B., Wu H., Liang Y., Ma P. X. (2018). Injectable antibacterial conductive nanocomposite cryogels with rapid shape recovery for noncompressible hemorrhage and wound healing. *Nature Communications*.

[12] Pedrotty D. M., Kuzmenko V., Karabulut E. (2019). Three-dimensional printed biopatches with conductive ink facilitate cardiac conduction when applied to disrupted myocardium. *Circulation. Arrhythmia and Electrophysiology*.

[13] Reddy S., Xiao Q., Liu H. (2019). Bionanotube/poly(3,4-ethylenedioxythiophene) nanohybrid as an electrode for the neural Interface and dopamine sensor. *ACS Applied Materials & Interfaces*.

[14] Scarano A., Carinci F., Piattelli A. (2009). Lip augmentation with a new filler (agarose gel): a 3-year follow-up study. *Oral Surgery, Oral Medicine, Oral Pathology, Oral Radiology, and Endodontics*.

[15] Gu Y., Tabata Y., Kawakami Y. (2017). Development of a new method to induce angiogenesis at subcutaneous site of Streptozotocin-induced diabetic rats for islet transplantation. *Cell Transplantation*.

[16] Varoni E., Tschon M., Palazzo B., Nitti P., Martini L., Rimondini L. (2012). Agarose gel as biomaterial or scaffold for implantation surgery: characterization, histological and histomorphometric study on soft tissue response. *Connective Tissue Research*.

[17] Fernandez-Cossio S., Leon-Mateos A., Sampedro F. G., Oreja M. T. (2007). Biocompatibility of agarose gel as a dermal filler: histologic evaluation of subcutaneous implants. *Plastic and Reconstructive Surgery*.

[18] Xu X. L., Lou J., Tang T. (2005). Evaluation of different scaffolds for BMP-2 genetic orthopedic tissue engineering. *Journal of Biomedical Materials Research Part B: Applied Biomaterials*.

[19] Iafisco M., Varoni E., Battistella E. (2010). The cooperative effect of size and crystallinity degree on the resorption of biomimetic hydroxyapatite for soft tissue augmentation. *The International Journal of Artificial Organs*.

[20] Rossi F., Santoro M., Casalini T., Veglianese P., Masi M., Perale G. (2011). Characterization and degradation behavior of agar-carbomer based hydrogels for drug delivery applications: solute effect. *International Journal of Molecular Sciences*.

[21] Stokols S., Tuszynski M. H. (2004). The fabrication and characterization of linearly oriented nerve guidance scaffolds for spinal cord injury. *Biomaterials*.

[22] Stokols S., Tuszynski M. H. (2006). Freeze-dried agarose scaffolds with uniaxial channels stimulate and guide linear axonal growth following spinal cord injury. *Biomaterials*.

[23] Felfel R. M., Gideon-Adeniyi M. J., Zakir Hossain K. M., Roberts G. A. F., Grant D. M. (2019). Structural, mechanical and swelling characteristics of 3D scaffolds from chitosan-agarose blends. *Carbohydrate Polymers*.

[24] Vadivelu R., Kashaninejad N., Sreejith K. R., Bhattacharjee R., Cock I., Nguyen N. T. (2018). Cryoprotectant-free freezing of cells using liquid marbles filled with hydrogel. *ACS Applied Materials & Interfaces*.

[25] Highley C. B., Song K. H., Daly A. C., Burdick J. A. (2019). Jammed microgel inks for 3D printing applications. *Advanced Science*.

[26] Zeng Q., Han Y., Li H., Chang J. (2015). Design of a thermosensitive bioglass/agarose–alginate composite hydrogel for chronic wound healing. *Journal of Materials Chemistry B*.

[27] Zhu Y., Kong L., Farhadi F. (2019). An injectable continuous stratified structurally and functionally biomimetic construct for enhancing osteochondral regeneration. *Biomaterials*.

[28] Paris J. L., Lafuente-Gomez N., Cabanas M. V., Roman J., Pena J., Vallet-Regi M. (2019). Fabrication of a nanoparticle-containing 3D porous bone scaffold with proangiogenic and antibacterial properties. *Acta Biomaterialia*.

[29] Zucca P., Fernandez-Lafuente R., Sanjust E. (2016). Agarose and its derivatives as supports for enzyme immobilization. *Molecules*.

[30] Zhang H., Patel A., Gaharwar A. K. (2013). Hyperbranched polyester hydrogels with controlled drug release and cell adhesion properties. *Biomacromolecules*.

[31] Atoufi Z., Zarrintaj P., Motlagh G. H., Amiri A., Bagher Z., Kamrava S. K. (2017). A novel bio electro active alginate-aniline tetramer/ agarose scaffold for tissue engineering: synthesis, characterization, drug release and cell culture study. *Journal of Biomaterials Science, Polymer Edition*.

[32] Sadeghi M., Hosseinzadeh H. (2013). Synthesis and properties of collagen-g-poly(sodium acrylate-co-2-hydroxyethylacrylate) superabsorbent hydrogels. *Brazilian Journal of Chemical Engineering*.

[33] Wang C., Pan Z. Z., Lv W. (2019). A directional strain sensor based on anisotropic microhoneycomb cellulose nanofiber-carbon nanotube hybrid aerogels prepared by unidirectional freeze drying. *Small*.

[34] Atoufi Z., Zarrintaj P., Motlagh G. H., Amiri A., Bagher Z., Kamrava S. K. (2017). A novel bio electro active alginate-aniline tetramer/agarose scaffold for tissue engineering: synthesis, characterization, drug release and cell culture study. *Journal of Biomaterials Science, Polymer Edition*.

[35] Lee S. J., Zhu W., Nowicki M. (2018). 3D printing nano conductive multi-walled carbon nanotube scaffolds for nerve regeneration. *Journal of Neural Engineering*.

[36] Alike Y., Yushan M., Keremu A. (2019). Application of custom anatomy-based nerve conduits on rabbit sciatic nerve defects: \*in vitro* and *in vivo* evaluations. *Neural Regeneration Research*.

[37] Wu Y., Wang L., Guo B., Ma P. X. (2017). Interwoven aligned conductive nanofiber yarn/hydrogel composite scaffolds for engineered 3D cardiac anisotropy. *ACS Nano*.

[38] Wang L., Wu Y., Hu T., Ma P. X., Guo B. (2019). Aligned conductive core-shell biomimetic scaffolds based on nanofiber yarns/hydrogel for enhanced 3D neurite outgrowth alignment and elongation. *Acta Biomaterialia*.

[39] Bozkurt A., Lassner F., O'Dey D. (2012). The role of microstructured and interconnected pore channels in a collagen-based nerve guide on axonal regeneration in peripheral nerves. *Biomaterials*.

[40] Dong R., Ma P. X., Guo B. (2020). Conductive biomaterials for muscle tissue engineering. *Biomaterials*.

[41] Sun Y., Quan Q., Meng H. (2019). Enhanced neurite outgrowth on a multiblock conductive nerve scaffold with self-powered electrical stimulation. *Advanced Healthcare Materials*.

[42] Haastert-Talini K., Grothe C. (2013). Electrical stimulation for promoting peripheral nerve regeneration. *International Review of Neurobiology*.

[43] Balint R., Cassidy N. J., Cartmell S. H. (2014). Conductive polymers: towards a smart biomaterial for tissue engineering. *Acta Biomaterialia*.

[44] Al-Majed A. A., Neumann C. M., Brushart T. M., Gordon T. (2000). Brief electrical stimulation promotes the speed and accuracy of motor axonal regeneration. *The Journal of Neuroscience*.

[45] Brushart T. M., Hoffman P. N., Royall R. M., Murinson B. B., Witzel C., Gordon T. (2002). Electrical stimulation promotes motoneuron regeneration without increasing its speed or conditioning the neuron. *The Journal of Neuroscience*.

[46] Brushart T. M., Jari R., Verge V., Rohde C., Gordon T. (2005). Electrical stimulation restores the specificity of sensory axon regeneration. *Experimental Neurology*.

[47] Asensio-Pinilla E., Udina E., Jaramillo J., Navarro X. (2009). Electrical stimulation combined with exercise increase axonal regeneration after peripheral nerve injury. *Experimental Neurology*.

[48] Gordon T., Brushart T. M., Chan K. M. (2008). Augmenting nerve regeneration with electrical stimulation. *Neurological Research*.

[49] Haastert-Talini K., Schmitte R., Korte N., Klode D., Ratzka A., Grothe C. (2011). Electrical stimulation accelerates axonal and functional peripheral nerve regeneration across long gaps. *Journal of Neurotrauma*.

[50] Singh B., Xu Q. G., Franz C. K. (2012). Accelerated axon outgrowth, guidance, and target reinnervation across nerve transection gaps following a brief electrical stimulation paradigm. *Journal of Neurosurgery*.

[51] Hsu C. C., Serio A., Amdursky N., Besnard C., Stevens M. M. (2018). Fabrication of Hemin-doped serum albumin-based fibrous scaffolds for neural tissue engineering applications. *ACS Applied Materials & Interfaces*.

[52] Liu X., Miller A. L., Park S. (2017). Functionalized carbon nanotube and Graphene oxide embedded electrically conductive hydrogel synergistically stimulates nerve cell differentiation. *ACS Applied Materials & Interfaces*.

[53] Singh N., Chen J., Koziol K. K. (2016). Chitin and carbon nanotube composites as biocompatible scaffolds for neuron growth. *Nanoscale*.

[54] Chen X., Liu C., Huang Z. (2019). Preparation of carboxylic graphene oxide-composited polypyrrole conduits and their effect on sciatic nerve repair under electrical stimulation. *Journal of Biomedical Materials Research Part A*.

[55] Koppes A. N., Keating K. W., McGregor A. L. (2016). Robust neurite extension following exogenous electrical stimulation within single walled carbon nanotube-composite hydrogels. *Acta Biomaterialia*.

[56] Ghasemi-Mobarakeh L., Prabhakaran M. P., Morshed M. (2011). Application of conductive polymers, scaffolds and electrical stimulation for nerve tissue engineering. *Journal of Tissue Engineering and Regenerative Medicine*.

[57] Guo B., Ma P. X. (2018). Conducting polymers for tissue engineering. *Biomacromolecules*.

[58] Wu Y., Wang L., Guo B., Shao Y., Ma P. X. (2016). Electroactive biodegradable polyurethane significantly enhanced Schwann cells myelin gene expression and neurotrophin secretion for peripheral nerve tissue engineering. *Biomaterials*.

[59] Wang S., Sun C., Guan S. (2017). Chitosan/gelatin porous scaffolds assembled with conductive poly(3,4-ethylenedioxythiophene) nanoparticles for neural tissue engineering. *Journal of Materials Chemistry B*.

[60] Wu Y., Wang L., Hu T., Ma P. X., Guo B. (2018). Conductive micropatterned polyurethane films as tissue engineering scaffolds for Schwann cells and PC12 cells. *Journal of Colloid and Interface Science*.

[61] Kazemi Y., Ramezani Kakroodi A., Ameli A., Filleter T., Park C. B. (2018). Highly stretchable conductive thermoplastic vulcanizate/carbon nanotube nanocomposites with segregated structure, low percolation threshold and improved cyclic electromechanical performance. *Journal of Materials Chemistry C*.

[62] Sambyal P., Iqbal A., Hong J. (2019). Ultralight and mechanically robust Ti_3_C_2_T_x_ Hybrid aerogel reinforced by carbon nanotubes for electromagnetic interference shielding. *ACS Applied Materials & Interfaces*.

[63] Taylor I. M., Patel N. A., Freedman N. C., Castagnola E., Cui X. T. (2019). Direct in vivo electrochemical detection of resting dopamine using Poly(3,4-ethylenedioxythiophene)/Carbon Nanotube functionalized microelectrodes. *Analytical Chemistry*.

[64] Sulaiman O. A., Gordon T. (2000). Effects of short- and long-term Schwann cell denervation on peripheral nerve regeneration, myelination, and size. *Glia*.

[65] Ramón-Azcón J., Ahadian S., Estili M. (2013). Dielectrophoretically aligned carbon nanotubes to control electrical and mechanical properties of hydrogels to fabricate contractile muscle myofibers. *Advanced Materials*.

[66] Nel A., Xia T., Madler L., Li N. (2006). Toxic potential of materials at the nanolevel. *Science*.

[67] Yan H., Gong A., He H., Zhou J., Wei Y., Lv L. (2006). Adsorption of microcystins by carbon nanotubes. *Chemosphere*.

[68] Bai Y., Park I. S., Lee S. J. (2011). Aqueous dispersion of surfactant-modified multiwalled carbon nanotubes and their application as an antibacterial agent. *Carbon*.

[69] Shin S. R., Bae H., Cha J. M. (2012). Carbon nanotube reinforced hybrid microgels as scaffold materials for cell encapsulation. *ACS Nano*.

[70] Alonso G. J., Rivera J. L. A., Mendoza A. M. M., Mendez M. L. H. (2007). Effect of temperature and pH on swelling behavior of hydroxyethyl cellullose-acrylamide hydrogel. *e-Polymers*.

[71] Liem R. K. (1990). Neuronal intermediate filaments. *Current Opinion in Cell Biology*.

[72] Watanabe M., Nakamura Y., Michalak Z. (2019). Serum GFAP and neurofilament light as biomarkers of disease activity and disability in NMOSD. *Neurology*.

[73] Montgomery M., Ahadian S., Davenport Huyer L. (2017). Flexible shape-memory scaffold for minimally invasive delivery of functional tissues. *Nature Materials*.

[74] Loh Q. L., Choong C. (2013). Three-dimensional scaffolds for tissue engineering applications: role of porosity and pore size. *Tissue Engineering Part B: Reviews*.

